# Ultrasound-guided continuous thoracic paravertebral block alleviates postoperative delirium in elderly patients undergoing esophagectomy: A randomized controlled trial

**DOI:** 10.1097/MD.0000000000019896

**Published:** 2020-04-24

**Authors:** Liang Jin, Rui Yao, Lei Heng, Bo Pang, Fu-Guo Sun, Ying Shen, Jun-Feng Zhong, Pan-Pan Zhao, Cong-You Wu, Bei-Ping Li

**Affiliations:** a Department of Anesthesiology, The People's Hospital of Leshan, Leshan; b Department of Anesthesiology, The Affiliated Xuzhou City Hospital of Xuzhou Medical University; c Department of Anesthesiology, Xuzhou Tumor Hospital, Xuzhou; d Department of Anesthesiology, Sichuan Provincial Corps Hospital, Chinese People's Armed Police Forces, Leshan; e Jiangsu Province Key Laboratory of Anesthesiology, Xuzhou Medical University, Xuzhou; f Department of Anesthesiology, The People's Hospital of Shaoxing, Shaoxing; g Department of Anesthesiology, Xuzhou Central Hospital, Xuzhou, China.

**Keywords:** inflammation, pain, paravertebral block, postoperative delirium

## Abstract

**Background:**

Delirium is a common postoperative complication in older patients undergoing thoracic surgery and presages poor outcomes. Postoperative pain is an important factor in the progression of delirium. The purpose of this study was to test whether continuous thoracic paravertebral block (PVB), a more effective approach for analgesia, could decrease the incidence of delirium in elderly patients undergoing esophagectomy.

**Methods:**

A total of 180 geriatric patients undergoing esophagectomy were randomly divided into 2 groups and treated with PVB or patient-controlled analgesia (PCA). Perioperative plasma CRP, IL-1β, IL-6, and TNF-α levels were detected in all patients. Pain intensity was measured by a numerical rating scale. Delirium was assessed using the confusion assessment method.

**Results:**

The incidence of postoperative delirium was significantly lower in the PVB group than in the PCA group. Patients in the PVB group had lower plasma CRP, IL-1β, IL-6, and TNF-α levels and less pain when coughing after surgery.

**Conclusions:**

Ultrasound-guided continuous thoracic paravertebral block improved analgesia, reduced the inflammatory reaction and decreased the occurrence of delirium after surgery.

## Abstract 2

Supplemental Digital Content is available in the text

## 1 Introduction

Postoperative delirium (POD) is characterized by disturbances in attention, cognition, and level of consciousness and is associated with prolonged hospitalization, increased morbidity and mortality and long-term cognitive dysfunction.^[1,2]^ An epidemiological study revealed that POD occurs in 11% to 51% of patients after surgery. Although the pathophysiology of POD remains largely unknown at the current time, neuroinflammation and pain, in particular, are cited as the main causes of POD.^[3,4]^

Neuroinflammation and pain are interrelated and reinforce each other. Postoperative pain may be an important factor affecting POD.^[5]^ A recent study showed that satisfactory analgesia can decrease the incidence of POD following hip or knee replacement surgery in geriatric patients.^[6]^ Pain has been shown to influence inflammatory processes in the brain. Animal studies have indicated that pain mediates neuroinflammation, triggering the activation of neurogliocytes and the concurrent endogenous production of pro-inflammatory cytokines.^[7,8]^ Increased expression of pro-inflammatory cytokines results in performance deficits of neuronal function, leading to POD.^[9]^

The analgesic effect of a paravertebral block (PVB) was likened to that of an epidural block and was found to be better than that of patient-controlled analgesia (PCA). PVB can effectively control acute postoperative pain with fewer side effects, such as nausea and vomiting, extradural hematoma, pulmonary complications, hypotension and urinary retention.^[10]^ Therefore, this prospective, randomized study was conducted to investigate the effect of PVB on POD incidence in geriatric patients undergoing esophagectomy. The effect of PVB on pro-inflammatory cytokine expression and the numerical rating scale (NRS) score for pain were also assessed.

## 2 Methods

### 2.1 Study design and participants

We performed a prospective, randomized parallel controlled trial registered with the Chinese Clinical Trial Register (ChiCTR-IOR-17013006). The study protocol was approved by the Ethics Committee of each participating center. Written informed consent was obtained from all patients. The inclusion criteria were patients aged 65 to 75 years who underwent elective esophagectomy for stage III and IV esophageal cancer. Patients who met any of the following criteria were excluded: allergic reactions to local anesthetic; brain injury or neurosurgery; cardiovascular or cerebrovascular disease; chronic obstructive pulmonary disease; neurological or psychiatric disorders; drug and alcohol abuse; hepatic and/or kidney dysfunction; BMI > 35 kg/m^2^; and inability to communicate. The included patients were randomized to receive ultrasound-guided continuous thoracic PVB before the induction of anesthesia or patient-controlled analgesia (PCA) at the end of the operation. The randomization was performed using an online randomization tool (http://www.randomization.com).

### 2.2 Anesthesia and operation protocol

All patients were intravenously administered scopolamine 0.3 mg (Shanghai Harvest Pharmaceutical Co., Ltd., Shanghai, China). Anesthesia was induced with 0.1 mg/kg midazolam (Nhwa Pharma. Corporation, Xuzhou, Jiangsu, China), 2 mg/kg propofol (Sichuan Guorui Pharmaceutical Co., Ltd, Leshan, Sichuan, China), 0.6 μg/kg sufentanil (Yichang Humanwell Pharmaceutical Co., Ltd, Yichang, Hubei, China), and 0.2 mg/kg cisatracurium (Jiangsu Hengrui Medicine Co., Ltd, Lianyugang, Jiangsu, China). Anesthesia was maintained with remifentanil (Yichang Humanwell) 0.1 to 0.2 μg/kg/min and propofol, with the propofol infusion rate adjusted to maintain the target Bispectral Index Score at 40–60. The tidal volume was adjusted to 8 ml/kg with a ventilatory frequency of 8 to 12 beats/min to maintain an end-tidal CO_2_ pressure (PETCO_2_) level of 30 to 40 mmHg. Heart rate, blood pressure and peripheral oxygen saturation (SpO_2_) were continuously recorded. All patients were intravenously administered 0.4 μg/kg sufentanil again when the thoracic incision was closed. Sufentanil and tropisetron were used for patient-controlled analgesia (PCA) within 48 hours post-surgery (continuous infusion with sufentanil 0.05 μg/kg/h and a bolus dose with sufentanil 0.03 μg/kg and a lock time of 15 minutes). A numerical rating scale (NRS) score from 0 to 10 was used to assess pain in all patients at rest and coughing for two days after surgery. Surgeons performed all esophagectomies via posterolateral thoracotomy. A single chest drain was placed in the sixth intercostal space in the mid-axillary line. The chest drain was removed 48 hours after surgery according to the volume of drainage and lung recruitment.

### 2.3 Ultrasound-guided continuous thoracic paravertebral block technique

**Figure 1:**
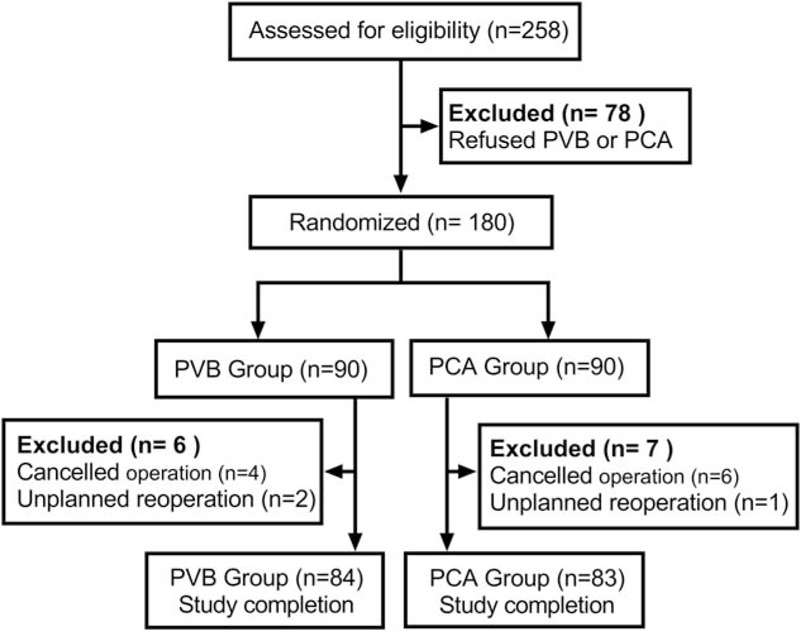
Enrollment flowchart. Flow of participants through each stage of our randomized trial. PVB = thoracic paravertebral block, PCA = patient-controlled analgesia (guidelines flow diagram).

After placing the patient in the lateral decubitus position, the anesthesiologist discerned the puncture site with a linear 5 to 10 MHz ultrasound probe (LOGIQe, GE Healthcare, Waukesha, WI) as follows: after distinguishing the targeted transverse process from the junction of the ribs on the horizontal plane, the cranial end of the transverse process was marked on the skin as the puncture site on the sagittal plane. After standard skin disinfection, a 17G Tuohy needle (Henan Tuoren Medical Device Co., Ltd., Xinxiang, Henan, China) was inserted perpendicularly or slightly caudally into the paravertebral space at the T4 or T5 level (Fig. 1A). A 20G catheter (Henan Tuoren Medical Device Co., Ltd., Xinxiang, Henan, China) was inserted up to the needle tip through the Tuohy needle, and then the Tuohy needle was withdrawn. The catheter was fixed to the skin and connected to a syringe. When aspiration demonstrated the absence of air or blood, 15 to 20 mL 0.375% ropivacaine and 10 μg sufentanil were injected into unilateral blocks before skin incision. Ten milliliters of 0.375% ropivacaine and 10 μg of sufentanil were injected through the catheter every 6 hours. The catheter was withdrawn 48 hours after the operation.

### 2.4 Delirium and secondary outcome assessment

Each patient was interviewed in the first 4 days after surgery. The prevalences of pulmonary atelectasis, nausea and pruritis were evaluated and recorded. The diagnosis of atelectasis was dependent on CT scans. Sedation or agitation was assessed using the Richmond Agitation Sedation Scale before the delirium assessment. The assessment of delirium was performed in the first 4 days after surgery between 8:00 AM and 10:00 AM. Delirium was diagnosed according to the confusion assessment method. The Chinese version of the confusion assessment method has been proven to have good reliability and validity with use in the Chinese elderly population^11^.

### 2.5 Laboratory measurements

Blood samples were collected at the following time points: preoperation (T1) and 24 hours (T2), 48 hours (T3) and 72 hours (T4) postsurgery. Plasma samples were centrifuged at 4000 rpm for 10 minutes and stored at −80°C. Plasma IL-1β, IL-6, TNF-α, and CRP levels were quantified using commercial enzyme-linked immunosorbent assay (ELISA) kits (Nanjing Jiancheng Biological Project Company, Nanjing, China). Biomarker standards and samples were added to the wells of assay plates and incubated for 1 hour at 37°C. The standard diluent was added to the blank wells. The horseradish peroxidase-conjugated antibody (0.1 mL) was added to each well and incubated for 40 minutes at 37°C. Subsequently, the plates were washed 4 times with phosphate-buffered saline, and chromogen solution (0.1 mL) was added to each well. The plates were gently mixed and incubated for 20 minutes at 37°C in the dark. Then, stop solution (0.05 mL) was added to each well, and the optical density was determined at 450 nm using a microplate reader. The plasma concentrations of inflammatory biomarkers were calculated based on the standard curves generated using recombinant human biomarkers.

### 2.6 Statistical analysis

A previous study found that the POD incidence was 23% in elderly patients after noncardiac surgery.^[11]^ With significance set at 0.05 and power set at 0.9, the calculated sample size should not be less than 148 according to GPower 3.1.9.2. Statistical analysis was performed using SPSS 19.0 (SPSS, Chicago, IL). The normality of the distribution of the variables was measured by the Shapiro-Wilk test. All quantitative data were normally distributed and are presented as the means ± standard deviations. The NRS scores between the 2 groups were compared by repeated measures ANOVA. Intervention state was considered the inter-subject factor test, and time of evaluation was considered the intra-subject factor. Comparisons of other quantitative data between the two groups were performed using Student's *t* test. Qualitative variables were compared using the chi-square or Fisher exact test.

## 3 Result

### 3.1 Patient characteristics

**Figure 2:**
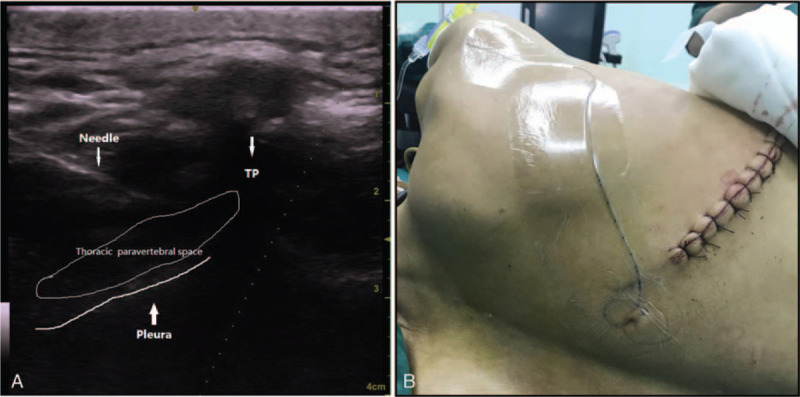
(A) Ultrasound image of in-plane needle advancement into the thoracic paravertebral space. TP = transverse process. (B) The catheter was inserted up to the thoracic paravertebral space and fixed to the skin.

A total of 180 patients completed the baseline assessment and were included in the trial from October 2017 to December 2018. The flowchart diagram is shown in Fig. 2. Six patients in the PVB group and seven patients in the PCA group were excluded before their follow-up appointment. No significant difference in basic demographic and other clinical characteristics was observed between the 2 groups (Table 1).

**Table 1:**
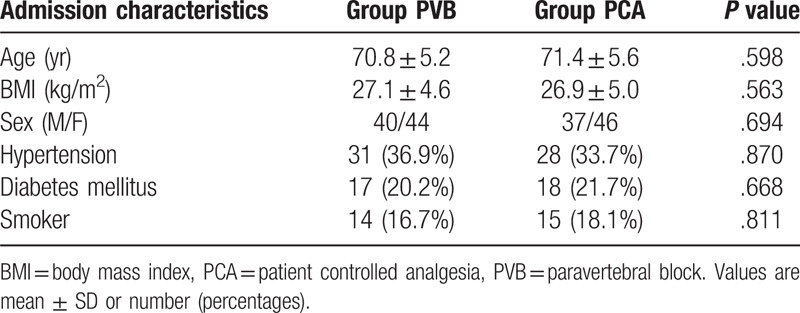
Demographic characteristics.

### 3.2 Secondary outcome

The total dose of propofol given was lower in the PVB group than in the PCA group (639 ± 91 mg vs 855 ± 110 mg; *P* = .008). The consumption of sufentanil was lower in the PVB group than in the PCA group from the end of surgery to 48 postoperative hours (80 ± 0 μg vs 156 ± 18 μg; *P* < .001). There were no differences in the consumption of remifentanil between the 2 groups during anesthesia. The prevalences of pulmonary atelectasis, nausea and pruritis were significantly lower in the PVB group than in the PCA group (atelectasis: 4.8% vs 14.5%; *P* = .033, nausea: 6.0% vs 19.3%; *P* = .009 and itchiness: 2.4% vs 10.8%; *P* = .028) (Table 2).

**Table 2:**
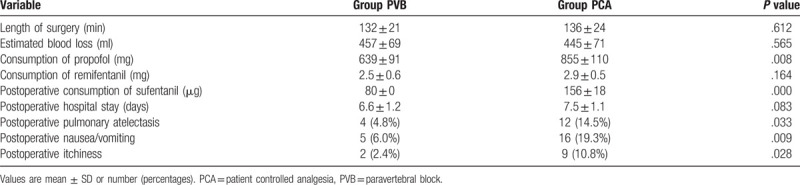
The characteristics of surgery and adverse.

### 3.3 Delirium assessment

**Figure 3:**
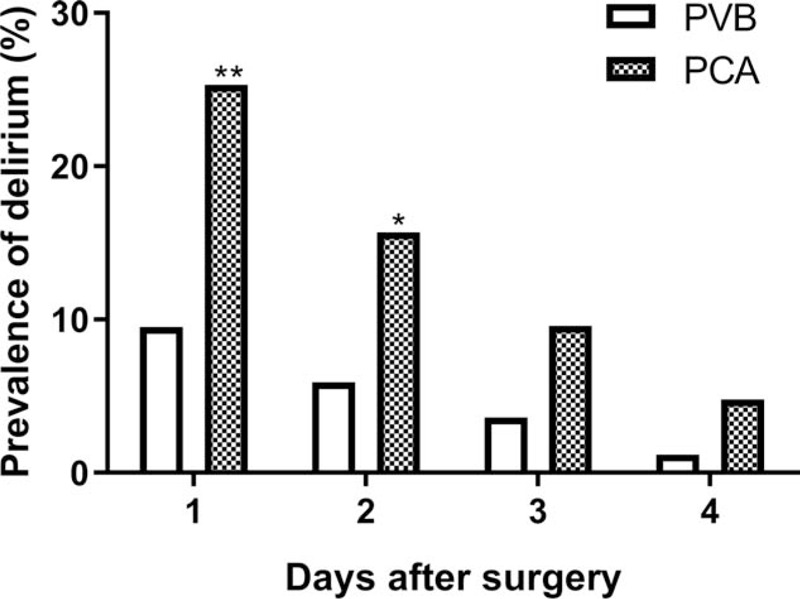
Daily prevalence of postoperative delirium. ^#^*P* < .05, ^##^*P* < .01, PVB group vs PCA group. PVB = thoracic paravertebral block, PCA = patient-controlled analgesia.

**Figure 4:**
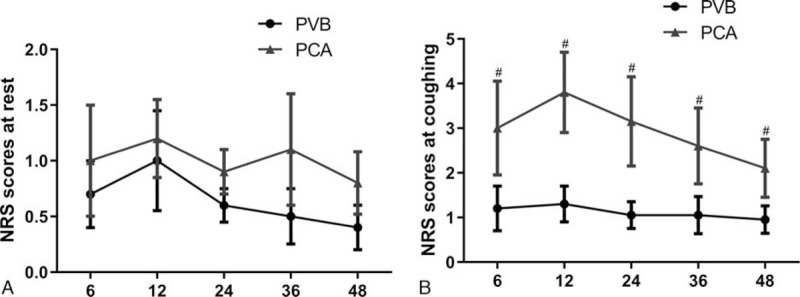
The mean and SD values of the numerical rating scale (NRS) scores in each group 6 to 48 hours after surgery. PVB = thoracic paravertebral block, PCA = patient-controlled analgesia. (A) There was no statistically significant time difference for NRS scores at rest between the 2 groups. (B) The NRS scores were lower in the PVB group than in the PCA group while coughing. ^#^*P* < .05, PVB group vs PCA group.

Eight out of 84 patients in the PVB group and 21 out of 83 patients in the PCA group met the diagnostic criteria for POD on the first postoperative day (9.5% vs 25.3%; *P* = .007). On the second postoperative day, 5 out of 84 patients in the PVB group and 13 out of 83 patients in the PCA group met the diagnostic criteria for POD (5.9% vs 15.7%; *P* = .043) (Fig. 3). The mean and SD values of the NRS scores in each group are shown in Fig. 4. The NRS scores were lower in the PVB group than in the PCA group when coughing (*P* < .05, Fig. 4B). There was no statistically significant time difference in the NRS scores at rest between the 2 groups (Fig. 4A).

### 3.4 Laboratory outcome

**Figure 5:**
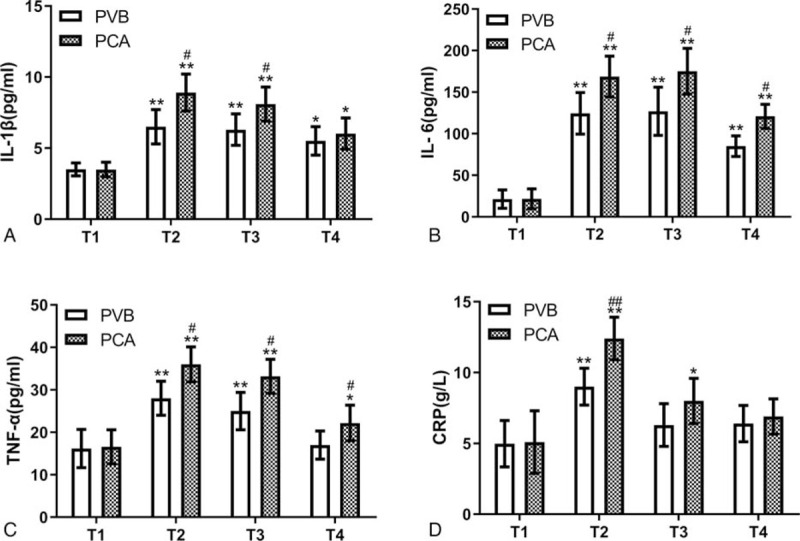
Plasma levels of IL-1β, IL-6, TNF-α and CRP before and after surgery in the PVB and PCA groups. ^∗^*P* < .05, ^∗∗^*P* < .01 vs baseline (T1), ^#^*P* < .05, ^##^*P* < .01 vs PVB group. PVB = thoracic paravertebral block, PCA = patient-controlled analgesia. T1, preoperation; T2, 24 hours after operation; T3, 48 hours after operation; T4, 72 hours after operation.

An ELISA for IL-1β showed that esophagectomy increased the levels of IL-1β after surgery in the PVB and PCA groups, but the levels in the PVB group were markedly lower than those in the PCA group at each time point postsurgery (*P* < .05, Fig. 5A). The changes in the levels of IL-6, TNF-α and CRP in the 2 groups were similar to those of IL-1β (Fig. 5B–D).

## 4 Discussion

In the present study, we assessed the effect of ultrasound-guided continuous thoracic paravertebral block (PVB) on postoperative delirium (POD) in geriatric patients. The patients who received PVB had a significantly lower incidence of POD as well as lower plasma levels of IL-1β, IL-6, TNF-α, and CRP postsurgery compared to the patients who received patient-controlled analgesia (PCA). Moreover, PVB provided more satisfactory analgesia than PCA and reduced postoperative complications. This was a randomized clinical trial that investigated the protective effect of PVB against POD and the possible underlying mechanism.

Increasing evidence demonstrates that insufficient postoperative analgesia likely plays a pivotal role in the development and progression of POD.^[12,13]^ Our data also showed that postoperative pain while coughing was not controlled effectively in patients with PCA, which could restrict coughing in patients. Upon further analysis, we found that NRS scores were higher in POD patients than in non-POD patients after surgery (see Fig. 1, Supplemental Content, which illustrates that the NRS scores were lower in non-POD patients than in POD patients while coughing). The results were broadly consistent with those of previous studies.^[14,15]^ Effective coughing and expectoration are very important to the recovery of lung function in patients undergoing thoracic surgery. Hypoxemia caused by pulmonary atelectasis may be a contributing factor to POD.^[16]^ We found that PVB provided satisfactory analgesia and reduced postoperative complications, including postoperative pulmonary atelectasis, which may be one of the mechanisms by which PVB alleviates POD. The inflammatory response also deserves our full attention. POD has been suggested to be related to inflammation.^[17,18]^ It is generally known that surgical injury activates the immune system, resulting in a peripheral inflammatory response. Pro-inflammatory cytokines, such as IL-1β, readily penetrate the blood-brain barrier (BBB) activating microglia and leading to neuroinflammation.^[19]^ In addition, pain is closely related to neuroinflammation.^[20]^ A recent study by Koyama et al^[21]^ described that acute postoperative pain exacerbates neuroinflammation and related delirium-like cognitive dysfunction in rats. Effective analgesia can lower stress levels and consequently reduce the systemic inflammatory response. In the current study, we found that PVB decreased the plasma concentrations of IL-1β, IL-6, TNF-α, and CRP while suppressing pain during the perioperative period. Further analysis revealed that the levels of pro-inflammatory cytokine levels in patients without POD were markedly lower than those in POD patients after surgery (Fig. 2, Supplemental Content, which shows plasma levels of IL-1β, IL-6, TNF-α, and CRP before and after surgery in patients who developed POD and who did not). This result suggests that the inflammatory response plays an important role in POD.

Unlike any postoperative intravenous analgesia, ultrasound-guided continuous thoracic PVB provides unintermittent and sufficient regional analgesia before starting the surgical procedure, which reduces the intraoperative consumption of anesthetic and opioid agents for postoperative analgesia. Fentanyl, morphine, and propofol have been reported as possible risk factors for POD.^[22–24]^ The present study showed that the intraoperative consumption of propofol and postoperative opioid agents were lower in the PVB group than in the PCA group. The subgroup analysis based on POD showed that the postoperative consumption of opioid agents in patients without POD was markedly lower than that in POD patients (Fig. 3, Supplemental Content, which shows the postoperative consumption of sufentanil in patients who developed POD and those who did not). The incidence of opioid-related complications in the PVB group was significantly lower than that in the PCA group. Decreasing the consumption of anesthetics and improving comfort may be another mechanism by which PVB alleviates POD.

This study had several limitations. First, double blinding was not used in the experiment. The catheter could be detected after ultrasound-guided thoracic paravertebral catheterization in the PVB group. Second, indwelling catheters may cause some inconvenience to the patients. The blockage effect of new long-term local anesthetics that are in development is expected to last for dozens of hours after a single injection. Finally, inflammatory mediators in the plasma directly reflect systemic inflammation rather than neuroinflammation. Cerebrospinal fluid may be more suitable than plasma for the evaluation of neuroinflammation but its use is restricted by ethical issues. Nevertheless, animal studies indicated that peripheral inflammatory cytokines could penetrate the BBB to activate neurogliocytes.^[25]^

In conclusion, our data demonstrated that ultrasound-guided continuous thoracic PVB alleviated POD in geriatric patients. The mechanisms by which PVB alleviates POD may be related to anti-inflammatory effects and decreased consumption of anesthetics in geriatric patients undergoing esophagectomy. Future studies with large sample sizes are needed to confirm the preventive effect of thoracic PVB against POD.

## Author contributions

**Conceptualization:** Congyou Wu, Bei-Ping Li.

**Data curation:** Ying Shen.

**Formal analysis:** Rui Yao, Lei Heng, Bo Pang, Ying Shen.

**Funding acquisition:** Congyou Wu, Bei-Ping Li.

**Investigation:** Liang Jin, Rui Yao, Lei Heng, Jun-Feng Zhong, Pan-Pan Zhao.

**Methodology:** Liang Jin, Rui Yao.

**Project administration:** Liang Jin, Rui Yao, Lei Heng, Fu-Guo Sun, Jun-Feng Zhong, Pan-Pan Zhao.

**Resources:** Fu-Guo Sun.

**Software:** Fu-Guo Sun, Ying Shen.

**Supervision:** Ying Shen, Bei-Ping Li.

**Validation:** Ying Shen.

**Writing – original draft:** Liang Jin, Rui Yao.

**Writing – review & editing:** Lei Heng, Bo Pang.

## Supplementary Material

Supplemental Digital Content

## Supplementary Material

Supplemental Digital Content

## Supplementary Material

Supplemental Digital Content
