# Supplementary material for: Ultrasound-guided continuous thoracic paravertebral block alleviates postoperative delirium in elderly patients undergoing esophagectomy: A randomized controlled trial
Source: Medicine (Baltimore). 2020 Apr 24;99(17):e19896. doi: 10.1097/MD.0000000000019896 (PMC7440095; doi:10.1097/MD.0000000000019896)

**Figure 1.** Numerical rating scale (NRS) scores in patients who developed postoperative delirium (POD) and who did not (no POD). (**A**) The NRS scores were less in no POD patients than in POD patients at rest 36 hours after surgery. There was no statistically significant time difference for NRS scores at other time points between the two groups. (**B**) The NRS scores were less in no POD patients than in POD patients at coughing. ^*^*P* < 0.05, no POD vs POD group.


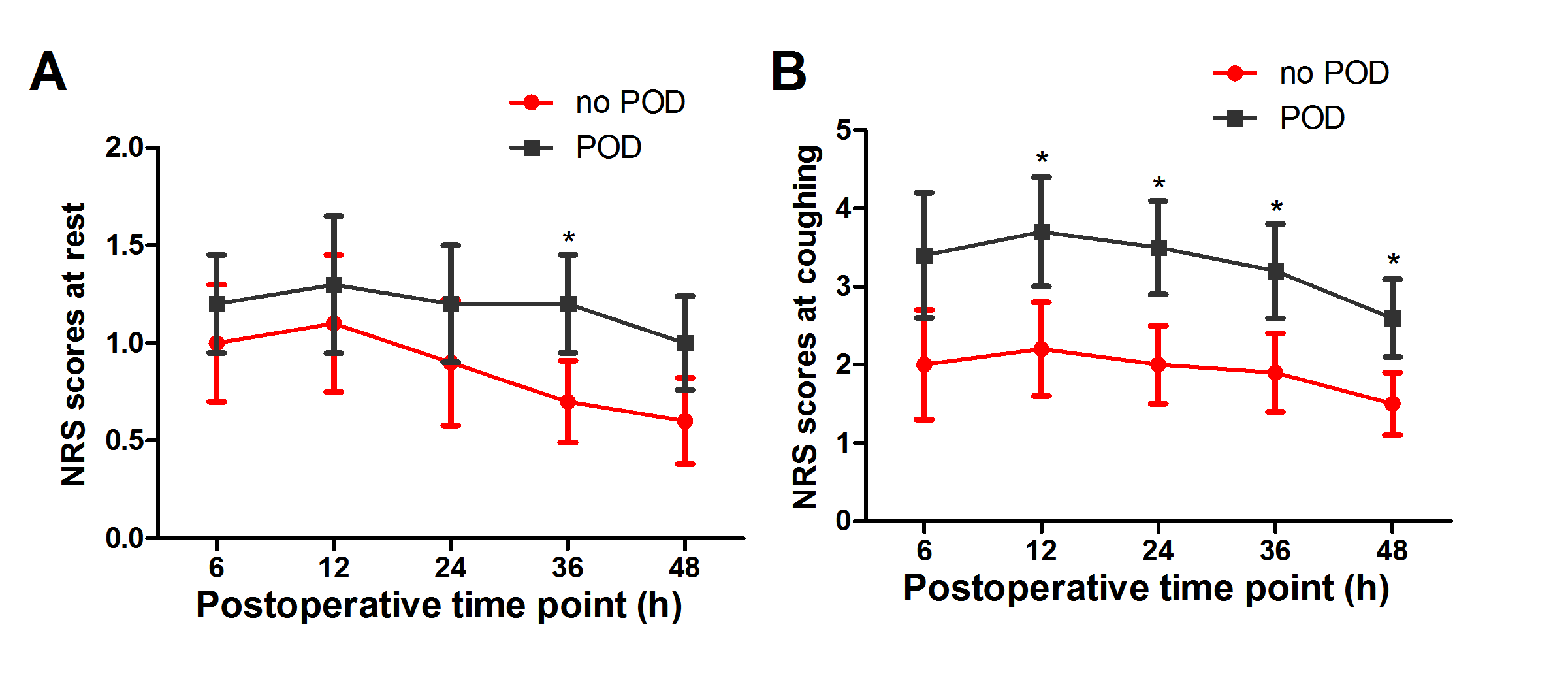

Supplement: Supplemental Digital Content [file medi-99-e19896-s001.docx]
