# Supplementary material for: Ultrasound-guided continuous thoracic paravertebral block alleviates postoperative delirium in elderly patients undergoing esophagectomy: A randomized controlled trial
Source: Medicine (Baltimore). 2020 Apr 24;99(17):e19896. doi: 10.1097/MD.0000000000019896 (PMC7440095; doi:10.1097/MD.0000000000019896)

**Figure 2.** Plasma levels of IL-1β, IL-6, TNF-α and CRP before and after surgery in patients who developed postoperative delirium (POD) and who did not (no POD). The cytokine levels in no POD patients were markedly lower than that in POD patients after surgery. ^*^*P* < 0.05, versus baseline (T1), ^#^*P* < 0.05, no POD vs POD group. T1, pre-operation; T2, 24 hours after operation; T3, 48 hours after operation; T4, 72 hours after operation.


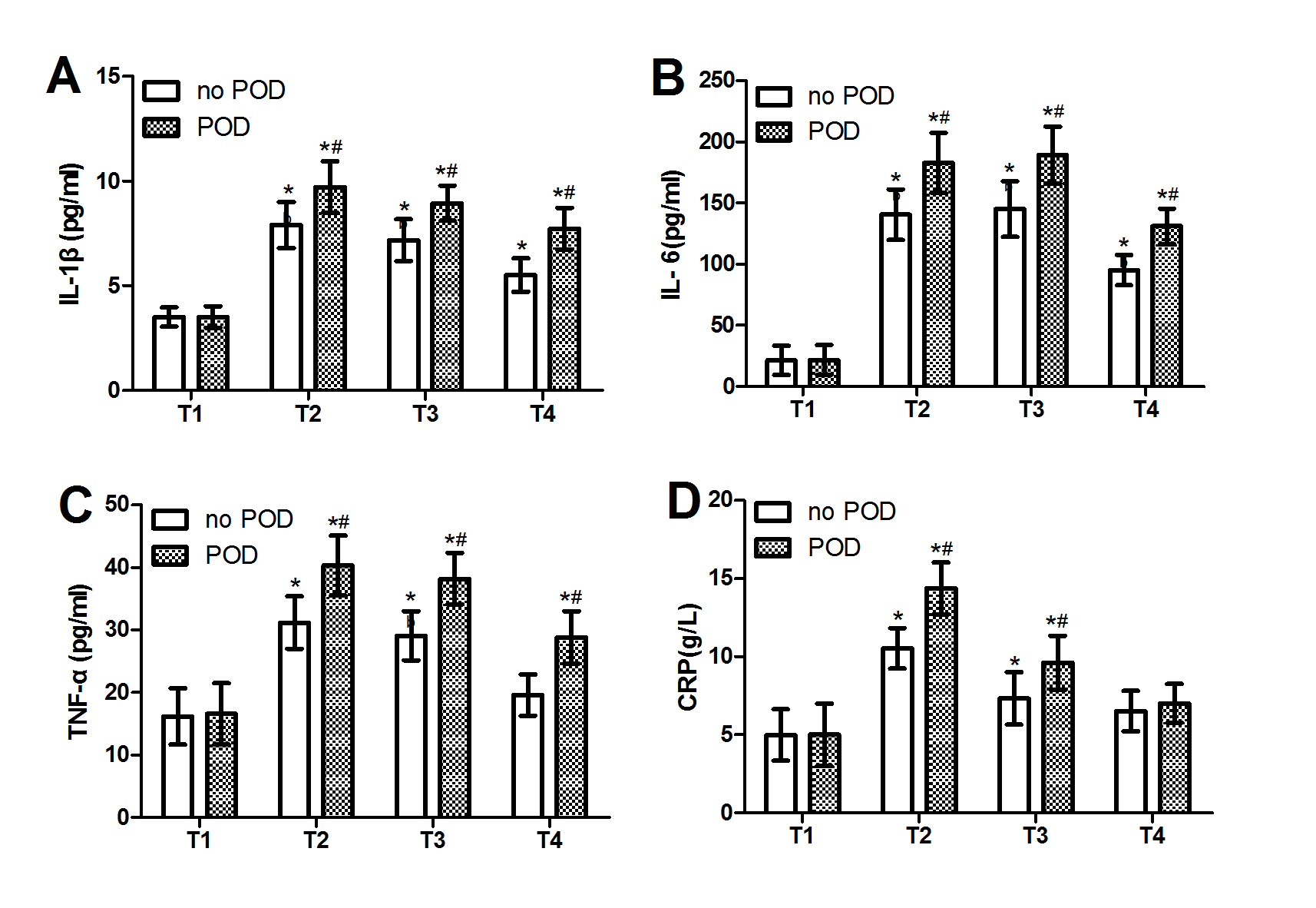

Supplement: Supplemental Digital Content [file medi-99-e19896-s002.docx]
