# Supplementary material for: Ultrasound-guided continuous thoracic paravertebral block alleviates postoperative delirium in elderly patients undergoing esophagectomy: A randomized controlled trial
Source: Medicine (Baltimore). 2020 Apr 24;99(17):e19896. doi: 10.1097/MD.0000000000019896 (PMC7440095; doi:10.1097/MD.0000000000019896)

**Figure 3.** Postoperative consumption of sufentanil in patients who developed postoperative delirium (POD) and who did not (no POD). ^#^*P* = 0.0025, no POD vs POD group.


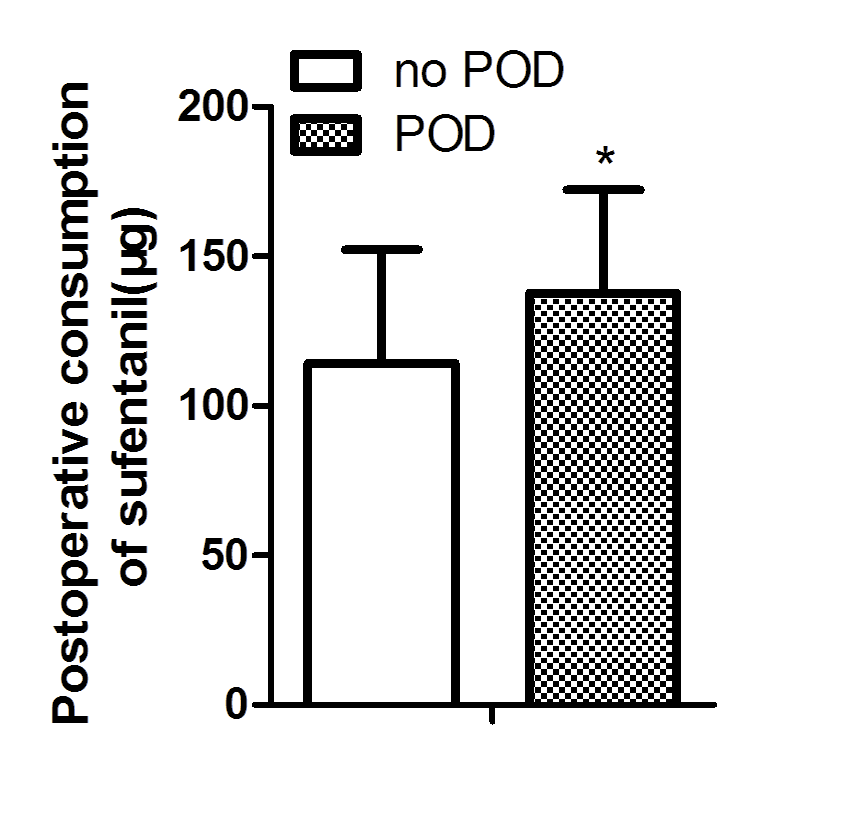

Supplement: Supplemental Digital Content [file medi-99-e19896-s003.docx]
